# The synergistic impact of low-carbon and innovative city construction on carbon intensity

**DOI:** 10.1186/s13021-026-00482-w

**Published:** 2026-06-29

**Authors:** Yan Zhang, Jiekuan Zhang

**Affiliations:** School of Tourism Management, Guilin Tourism University, Guilin, China

**Keywords:** Low-carbon city, Innovative city, Carbon intensity, Difference-in-differences model

## Abstract

Against the backdrop of global climate change and sustainable development, cities, as major sources of carbon emissions, play a pivotal role in achieving carbon neutrality goals. This study examines the synergistic impact of innovative city and low-carbon city pilots on carbon intensity, using panel data from 272 Chinese cities. The findings reveal that low-carbon and innovative city construction (LCICC) significantly reduces carbon intensity, primarily through technological advances, industrial upgrading, and green finance. However, the carbon intensity reduction is only significant in central regions and resource-based cities. Conversely, LCICC significantly increases carbon intensity in cities characterized by lower administrative status and non-resource-dependent economic structures. This study not only enriches the theory of urban transformation and provides new perspectives for carbon reduction research but also offers the first empirical evidence of synergistic emission reduction effects from the simultaneous implementation of innovation and low-carbon city pilots. These findings provide a replicable analytical framework for other developing economies pursuing dual policy pathways toward carbon neutrality.

## Introduction

In recent years, the construction of innovative cities and low-carbon cities, as core strategies for urban transformation, have generated changes across economic, social, and environmental dimensions, ranging from green logistics efficiency [1] and green development [2] to energy efficiency [3], ecological efficiency [4, 5], total factor productivity [6], and innovation efficiency [7]. Although both innovative city pilots and low-carbon city pilots are highly relevant to carbon intensity reduction, existing studies have typically examined them in isolation. Innovative city construction primarily targets technological progress and industrial upgrading, which have been shown to enhance energy productivity and reduce emissions per unit of output [3, 8]. Low-carbon city construction imposes explicit carbon constraints and promotes energy structure optimization and carbon trading mechanisms [9, 10]. More importantly, these two policies exhibit substantial temporal and spatial overlap, offering a quasi-experimental setting to examine how innovation-driven and low-carbon transformations jointly influence carbon intensity—an issue that remains underexplored. Other urban pilot programs, such as smart cities, sponge cities, or circular economy pilots, either focus on different environmental dimensions (e.g. water management or waste recycling) or were implemented later than the sample period of this study, making them less suitable for investigating the synergistic effects on carbon intensity [11]. With the intensification of global climate change, cities, as the main sources of carbon emissions, play a crucial role in achieving carbon neutrality targets. Therefore, a growing body of literature has explored the impact of low-carbon or innovative city construction on carbon emissions [12–14]. However, existing studies mostly focus on single transformation policies, lacking in-depth exploration of the comprehensive transformation effects and still insufficient in studying policy synergy effects.

With the acceleration of urbanization and the growth of energy demand, the pressure of urban carbon emissions continues to increase, urgently requiring scientific evaluation and policy optimization to enhance the carbon reduction effects of low-carbon and innovative city construction (LCICC). Currently, literature pays more attention to total carbon emissions and to some extent neglects carbon intensity, an important indicator of low-carbon development. Developing nations face a unique climate challenge: they must pursue aggressive emission reductions while compensating for their late industrialization and ongoing development needs. In this context, prioritizing carbon intensity management emerges as a crucial strategy for these economies [15]. Current academic investigations into the urban transformation-emission nexus, while valuable, have predominantly focused on technological innovation and industrial restructuring mechanisms [14, 16]. This research landscape reveals significant gaps, particularly in understanding the mediating roles of policy instruments and economic factors, including environmental governance frameworks and financial system development. Consequently, a comprehensive investigation into the mechanisms through which LCICC influences carbon intensity carries substantial academic and practical implications. Such research endeavors would contribute to advancing theoretical frameworks in sustainable urban studies while simultaneously offering evidence-based insights for policy formulation. Addressing the existing knowledge gaps, this study focuses on examining the multidimensional relationship between urban transformation processes and carbon intensity dynamics. Particular emphasis is placed on exploring the integrative potential between innovation-driven urban development strategies and low-carbon city initiatives, representing a critical area of inquiry with both theoretical relevance and policy applicability. Against this backdrop, the significance of our study lies in systematically quantifying the synergistic effect of dual-pilot policies, an issue that has profound implications for designing cost-effective urban transformation strategies in emerging economies, where fiscal resources and administrative capacity are often constrained.

This research adopts a quasi-experimental approach to systematically assess the relationship between LCICC and carbon intensity dynamics, utilizing China’s urban development trajectory as an empirical case study. The analytical framework employs the difference-in-differences (DID) methodology, leveraging the natural experiment created by China’s sequential policy initiatives - the innovative city pilot program initiated in 2008 followed by the low-carbon city pilot scheme in 2010 [17–18]. These staggered policy implementations provide robust research setting for examining urban transformation’s environmental impacts. Through comparative analysis of carbon intensity trajectories between treatment (pilot) and control (non-pilot) groups across pre- and post-intervention periods, the study effectively isolates the causal effects of urban transformation while mitigating potential confounding variables. Moreover, the DID method offers distinct advantages in addressing endogeneity concerns, particularly through its dual-dimensional comparison of temporal variations (pre- and post-policy implementation) and cross-sectional differences (between treatment and control groups), thereby enabling more precise estimation of urban transformation policy effects [19–20]. To further enhance the analytical depth, this investigation incorporates a comprehensive research framework that integrates mediating mechanism examination with spatial heterogeneity analysis.

The theoretical contributions of this study include the two facets: First, by systematically analyzing the impact mechanism of LCICC on carbon intensity, it enriches the theory of sustainable urban development, especially the research on the synergy between the construction of innovative cities and low-carbon cities, providing theoretical support for policymaking. Second, through the DID method and mechanism analysis, it reveals the multiple pathways of LCICC, providing a new theoretical perspective for understanding the dynamics and heterogeneity of policy effects and offering a Chinese case for global carbon emission reduction research. In terms of practical value, this study provides the following references for policymakers, firstly, to optimize the design of LCICC policies, enhance policy synergy effects, and help achieve dual carbon goals. Secondly, this research aims to establish an evidence-based framework for urban policy formulation, with particular emphasis on identifying effective pathways for carbon intensity reduction through three key mechanisms: technological advancement, industrial restructuring, and sustainable financial innovation. Thirdly, the study seeks to develop region-specific transformation strategies that account for the diverse developmental characteristics across different geographical areas. The findings are expected to contribute significantly to China’s dual carbon objectives (peak emissions and carbon neutrality) while simultaneously offering valuable insights for international urban sustainability practices, particularly for emerging economies undergoing similar urbanization processes.

## Theoretical background and hypothesis

As fundamental spatial entities driving regional progress, urban centers have emerged as pivotal hubs in the global innovation ecosystem. Within the context of intensifying inter-city competition and accelerating urbanization trends, urban development goals are increasingly defined through the discourse of innovation [21]. Through the construction of innovative cities, innovation elements are fully utilized, forming an innovation system that encompasses technology, institutions, and politics, thereby profoundly influencing urban economic growth and development. Studies have demonstrated that innovative city development contributes to reducing regional carbon emissions [17, 22], enhancing energy efficiency [3], and reducing industrial carbon emissions through technological innovation and industrial upgrading [8, 23]. Cities also account for the majority of global carbon dioxide emissions, making urban low-carbon transformation a core component of global low-carbon strategies [19], giving rise to the concept of low-carbon cities. Low-carbon cities aim to balance economic development and low-carbon practices through ecological planning, fostering sustainable economic and environmental development. The construction of low-carbon cities has been shown to reduce pollution emissions [3, 24], improve ecological efficiency [5], promote green growth [2], and directly reduce urban carbon emissions by optimizing energy structures, promoting clean energy, and implementing carbon trading policies [9, 10, 25].

Unlike carbon emissions, carbon intensity measures the efficiency of carbon emissions per unit of economic growth. Given its dual focus on carbon emissions and economic growth, carbon intensity has received increasing attention in assessing local low-carbon and sustainable development. For example, China’s carbon peak and carbon neutrality targets are set for 2030 and 2060, respectively. However, as early as the 2009 Copenhagen Climate Conference, China established ambitious carbon intensity reduction targets, mandating a 40%-45% decrease in CO₂ emissions per unit of GDP by 2020 relative to 2005 levels, with subsequent goals of achieving 60%-65% reduction by 2030. This policy framework has stimulated academic interest in examining the relationship between LCICC processes and carbon intensity dynamics. However, research in this specific area remains relatively underdeveloped compared to the more extensive body of literature focusing on absolute carbon emissions, highlighting a significant gap in current urban sustainability studies.

For instance, Wang and Zhou [26] provided evidence supporting the effectiveness of innovation-oriented urban policies, while Gao and Wang [13] and Liu et al. [19] established the positive environmental impacts of low-carbon city initiatives. However, it is crucial to recognize that LCICC is often multidimensional, not limited to innovative city construction, low-carbon city construction, or other single aspects. Some cities undergo dual transformations, combining innovation and low-carbon development [11]. For cities undergoing dual transformations, carbon reduction can be achieved through multiple pathways, including economic and industrial restructuring, social and lifestyle changes, technological innovation, and ecological transformation. The interaction of these multiple elements suggests that comprehensive LCICC can achieve substantially greater reductions in carbon intensity than any single-dimensional approach.

To understand why the joint implementation of innovative and low-carbon city policies can produce effects beyond their individual contributions, we propose three synergistic mechanisms. First, technology-push and technology-pull complementarity: innovative city construction acts as a technology-push mechanism, stimulating R&D in green technologies and energy efficiency [8, 27], while low-carbon city construction creates a technology-pull through carbon constraints and market incentives [9, 10]. Together, they accelerate low-carbon innovation adoption more effectively than either policy alone [11, 28]. Second, risk diversification and cost-sharing: a single-dimensional transformation carries significant risks. An innovation-only approach may fail due to technological lags, whereas a low-carbon-only approach may encounter industry resistance [29]. Dual implementation diversifies risks and pools central government funding, reducing marginal policy costs [30, 31]. More broadly, the effectiveness of dual-pilot policies depends on building consensus among multiple stakeholders, including central and local governments, firms, and the public, a process that collaborative governance frameworks explicitly address [32]. Third, policy signal reinforcement and green finance mobilization: dual designation sends a stronger credibility signal to private investors, reducing regulatory uncertainty and attracting green finance such as green bonds and ESG funds [33–34]. This reinforced signaling accelerates low-carbon technology deployment.

In summary, LCICC has become a crucial strategy for addressing global climate change and promoting sustainable development. Therefore, we propose the first hypothesis:

### Hypothesis 1

The simultaneous implementation of innovative and low-carbon city construction generates synergistic emission reduction effects that exceed the sum of individual policy impacts. Specifically, cities implementing both policies see a significantly larger reduction in carbon intensity than those implementing only one. This synergistic effect operates through three channels: technology-push-pull complementarity, risk-cost sharing, and reinforced policy signaling.

During LCICC, technological innovation serves as a key mechanism for reducing carbon intensity. Institutional economics and innovation system theory suggest that policy-driven urban transformation fosters R&D activity and technology diffusion [35–36]. LCICC facilitates this by optimizing policy environments, increasing funding, and promoting industry-university-research collaboration [18, 27]. Technological innovation reduces carbon intensity through two channels: improving energy efficiency and enabling low-carbon technology adoption [8, 37–38]. While prior studies have confirmed the emission-reducing role of green patents and renewable energy innovation [9, 39], the specific contribution of LCICC-induced innovation to carbon intensity remains underexplored. Thus, we propose:

### Hypothesis 2

LCICC significantly promotes technological innovation, thereby reducing regional carbon intensity.

Industrial restructuring is another critical pathway. Industrial structure theory indicates that shifting away from high-carbon industries and optimizing inter-sectoral coordination can lower emissions per unit of output [40]. LCICC accelerates this transition through policy guidance, technological retrofitting, and support for emerging low-carbon sectors [9, 41]. Two dimensions matter: industrial structure advancement (the rise of service-oriented sectors) and industrial structure rationalization (efficient, coordinated development among industries). Both have been shown to reduce carbon intensity [42–43]. However, evidence on how LCICC specifically drives these two dimensions remains limited. Therefore:

### Hypothesis 3

LCICC significantly promotes industrial structure upgrading, thereby reducing regional carbon intensity.

### Hypothesis 3a

LCICC significantly promotes industrial structure advancement, thereby reducing regional carbon intensity.

### Hypothesis 3b

LCICC significantly promotes industrial structure rationalization, thereby reducing regional carbon intensity.

Environmental regulation is often hypothesized to reduce carbon intensity by incentivizing green innovation and reallocating resources away from polluting sectors, as argued by the Porter Hypothesis [28]. LCICC could intensify regulation through stricter emission standards, carbon trading systems, and enhanced supervision [44–45]. However, the empirical evidence is mixed: some studies find positive effects, while others suggest that regulatory intensity may be diluted by competing local priorities such as economic growth. This tension makes the environmental regulation mechanism an open empirical question. Hence:

### Hypothesis 4

LCICC significantly enhances the intensity of environmental regulation, thereby reducing regional carbon intensity.

Green finance represents a market-based mechanism that aligns financial flows with low-carbon development. By channeling capital toward green projects through green credit, bonds, and investment funds, green finance reduces carbon intensity while supporting technological innovation and industrial upgrading [30, 33, 46]. LCICC is expected to promote green finance by creating policy signals and reducing information asymmetries [34, 47–48]. Yet, the extent to which LCICC leverages green finance as a transmission channel has received little direct empirical testing. We therefore propose:

### Hypothesis 5

LCICC significantly reduces regional carbon intensity by enhancing the level of green finance.

**Fig. 1:**
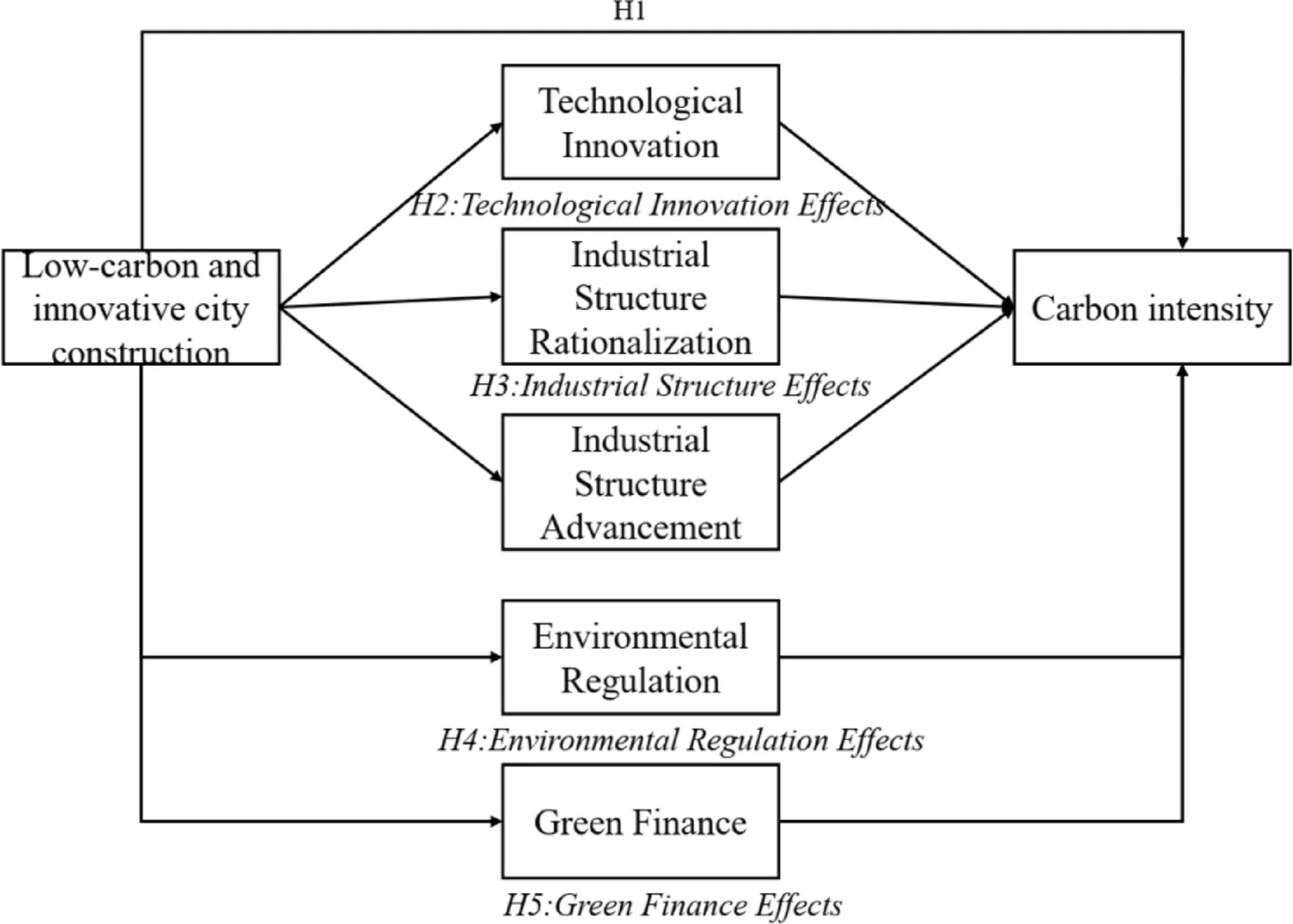
Theoretical framework

In summary, LCICC is expected to reduce regional carbon intensity through technological innovation effects, industrial structure effects, environmental regulation effects, and green finance effects. Figure 1 illustrates the main theoretical framework of this study.

## Methodologies

### Modelling

Taking advantage of the quasi-experimental design of LCICC policies (see the independent variable section), this study employs a time-varying difference-in-differences (DID) specification with two-way fixed effects.

1 $$\begin{aligned}carbon\_intensity_{i,t} & = \beta_{0} + \beta_{1}did_{i,t} + \sum \beta_{j}control_{i,t}\\ & \quad + \mu_{i} + \lambda_{t} + \varepsilon_{i,t} \end{aligned}$$

The subscripts *i* and *t* correspond to city-level entities and temporal dimensions, respectively. The key explanatory variable, *did*, functions as a binary indicator for LCICC policy implementation, while the vector control encompasses multiple covariates accounting for potential confounding factors. The model incorporates *µ*_*i*_ and *λ*_*t*_ as city-specific and time-specific fixed effects, respectively, with *ε*_*i, t*_ representing the stochastic error component. The coefficient *β*_*1*_ captures the net treatment effect of LCICC policies.

### Variable and measurement

#### Dependent variable: carbon intensity

Carbon intensity represents a crucial metric in environmental economics, quantifying the ratio of carbon dioxide emissions to economic output or energy input, conventionally expressed as CO_2_ emissions per unit of GDP. This relative measure serves as an important indicator of economic-environmental efficiency, distinguishing itself from absolute emission metrics by capturing the decoupling degree between economic growth and environmental impact. Unlike gross carbon emissions, which reflect total environmental burden, carbon intensity provides insights into the sustainability and resource efficiency of economic activities. Carbon intensity can change with policy and technological advancements, energy structure optimization, and economic restructuring [49–50]. In contemporary sustainability assessment frameworks, carbon intensity has emerged as a pivotal indicator of development quality, as its reduction reflects improved environmental-economic efficiency. This metric captures the essential balance between economic growth and ecological impact, where declining carbon intensity values typically result from three fundamental drivers: technological innovation, enhanced energy mix composition, and structural economic transformation. The measurement’s significance lies in its ability to simultaneously account for both environmental protection and economic performance, making it particularly valuable for evaluating sustainable development trajectories. Moreover, many countries and regions aim to decouple economic growth from carbon emissions by reducing carbon intensity, thereby promoting sustainable development [51]. Additionally, carbon intensity provides a relative standard that facilitates comparisons and cooperation among different countries and regions, jointly advancing global sustainable development [52]. The carbon emissions data are collected from the EDGAR for Chinese prefecture-level cities [53], while economic output metrics are obtained from the official *China City Statistical Yearbook*.

#### Independent variable: low-carbon and innovative city construction

The independent variable is a cross-term dummy variable (did) for innovative and low-carbon city construction pilots. In accordance with standard difference-in-differences methodology, the treatment indicator variable is assigned a value of 1 for all observation years following a city’s designation as either an innovative city or low-carbon city pilot, including the year of policy implementation. Conversely, the variable takes a value of 0 for all pre-treatment periods. Cities that were never designated as pilots during the sample period are assigned a value of 0 throughout. China has conducted multiple rounds of pilot programs from 2008 to 2013 and in 2018, resulting in 78 innovative pilot cities by 2019. The Chinese government implemented its low-carbon city initiative through three distinct phases, designating a total of 81 pilot cities across batches in 2010, 2012, and 2017. To capture the synergistic effects of LCICC policies, this research employs an interaction term constructed as the multiplicative product of innovative city and low-carbon city transformation indices. This methodological approach allows for the quantification of policy complementarity and joint effects, with the resulting product serving as the value for the cross-term treatment indicator (*did*) in the empirical analysis.

#### Mechanism variable

Technological innovation capacity is conventionally operationalized through patent-based metrics, particularly using the count of granted patents as a proxy indicator [36], with data extracted from the authoritative *China City Statistical Yearbook*. Industrial structure rationalization, a multidimensional concept, captures the level of industrial integration and the optimal allocation of production factors. This metric simultaneously reflects inter-industry coordination efficiency and resource allocation effectiveness. Following established methodologies, this research employs the Theil index approach [54] to quantify urban industrial structure rationalization, with the computational framework expressed as follows.

2 $$S = \sum_{i=1}^{3} (\frac{Y_{i}}{Y})\mathrm{ln}(\frac{Y_{i}}{L_{i}}\bigg/ \frac{Y}{L} ) $$

In this formulation, *S* serves as the quantitative measure of industrial structure rationalization, where *Y* represents sectoral output values and L corresponds to employment distribution across economic sectors. The subscript *i* enumerates the three major industrial classifications. The metric exhibits specific boundary conditions, with S = 0 indicating optimal industrial structure equilibrium, while any positive value reflects varying degrees of structural imbalance. The magnitude of *S* demonstrates a positive correlation with the extent of industrial structure irrationality, providing a continuous scale for assessing structural efficiency.

Industrial structure advancement reflects the service-oriented tendency of the overall economic system and is measured by the ratio of the added value of the tertiary industry to that of the secondary industry [55]. The data on added value and employment for the aforementioned industries are sourced from the *China City Statistical Yearbook*. The intensity of environmental regulation is quantified through textual analysis of government work reports, specifically measuring the relative frequency of environment-related terminology. This approach operates on the premise that the prevalence of such terms correlates with the prioritization of environmental governance in policy agendas, suggesting more comprehensive environmental management strategies at the local level [56]. The analysis encompasses a broad spectrum of environmental concepts, including but not limited to ecological preservation, pollution control, energy efficiency, emission mitigation, and air quality management. To capture the substantive policy emphasis rather than mere term frequency, this study employs a refined metric that calculates the proportion of sentences containing environmental terminology relative to the total report length. The dataset is constructed from systematically analyzed annual government work reports across prefecture-level municipalities, ensuring representative coverage of local environmental policy orientations.

Departing from conventional approaches that conceptualize green finance as a singular policy instrument [30, 46] or rely on isolated metrics like green credit [47], this research develops a multidimensional green finance index that integrates seven distinct yet interconnected dimensions: credit allocation, investment patterns, insurance products, bond markets, fiscal support, fund markets, and equity trading. Specifically, these dimensions are operationalized through: (a) environmental project credit allocation ratios, (b) pollution control investment-to-GDP ratios, (c) environmental liability insurance revenue shares, (d) green bond issuance proportions, (e) environmental protection expenditure ratios within fiscal budgets, (f) green fund market capitalization shares, and (g) carbon/energy/pollution rights trading volumes relative to total equity market activity. The comprehensive dataset is compiled from multiple authoritative sources, including national statistical bureaus, central bank reports, ministerial publications, and regional statistical yearbooks, complemented by municipal environmental bulletins and specialized statistical compendiums covering science, energy, finance, agriculture, industry, and tertiary sectors. To synthesize these diverse indicators into a unified metric, the study employs the entropy weighting method, which objectively determines indicator weights based on information content, thereby generating a robust composite measure of green finance development.

#### Control variables

To ensure the robustness and reliability of the empirical findings, Model (1) incorporates a comprehensive set of control variables informed by established literature on carbon emissions and intensity dynamics. Drawing on previous studies [57–60], the analysis accounts for six key dimensions: economic development level, foreign direct investment intensity, international trade exposure, population density, educational investment, and urbanization rate. These variables are operationalized through specific metrics: (a) economic output per capita for economic development, (b) the ratio of utilized foreign capital to GDP for foreign investment, (c) the trade-to-GDP ratio for foreign trade engagement, (d) population per square kilometer for demographic concentration, (e) the share of education expenditure in total fiscal spending for educational development, and (f) the urban population proportion for urbanization level. The dataset is compiled from authoritative sources, primarily the *China City Statistical Yearbook* and various municipal statistical yearbooks.

Considering data availability, the earliest data in this study is from 2005. Additionally, given the significant impact of the COVID-19 pandemic on China’s socio-economic conditions, especially the carbon emissions, after 2020 [50, 61], the data is truncated at 2019. Ultimately, panel data for 272 prefecture-level cities from 2005 to 2019 are obtained. It should be noted that not considering the latest data will not affect the DID estimation results, as the sample time interval already has sufficient pre- and post-event observations. The 272 sample cities include 56 innovative construction pilot cities and 64 low-carbon construction pilot cities. Given the differences in the magnitude of different variables, the outputs of technological innovation, population density, and per capita GDP are logarithmically transformed in the model. To account for price factors, per capita GDP is converted to real values based on 2005 constant prices. Table 1 presents the descriptive statistics of the variables.

**Table 1:** Descriptive statistics

Variable	Variable type	Mean	Std. Dev.	Min	Max
Carbon intensity	Dependent variable	2.534	2.354	0.043	25.717
LCICC	Independent variable	0.035	0.183	0	1
Technological innovation	Mechanism variable	3265.838	8710.960	0	166,609
Industrial structure rationalization		0.273	0.207	0.000	1.722
Industrial structure advancement		0.895	0.465	0.075	4.946
Environmental regulation		0.003	0.002	0	0.012
Green finance		0.295	0.098	0.054	0.624
Economy	Control variable	10.134	0.687	7.912	12.196
Foreign investment		0.018	0.019	0	0.200
Trading openness		0.190	0.373	0.000	7.846
Population		444.999	489.849	5.819	6729.494
Education		0.183	0.044	0.018	0.494
Urbanization		0.502	0.169	0.105	1

## Results and discussion

### Benchmark regression

Table 2 reports the benchmark regression results. Columns 1–3 show the impact of LCICC, innovative city construction, and low-carbon construction on carbon intensity, respectively. The empirical results demonstrate consistently negative coefficients across all transformation categories, suggesting that LCICC significantly reduces carbon intensity. Thus, Hypothesis 1 is supported. Additionally, both innovative city construction and low-carbon city construction negatively affect carbon intensity, but their impact is weaker than the cross-effect of innovative and low-carbon transformation. This indicates that LCICC generates a positive synergistic effect on carbon intensity reduction, and our analysis focuses on the cross-effect of urban innovation and low-carbon transformation. Consistent with previous findings that innovative city construction contributes to green growth [62], improves ecological efficiency [4], enhances energy efficiency [3], and reduces carbon emissions [22], and that low-carbon city construction improves ecological efficiency [5], promotes green growth [2], and reduces carbon emissions [25], comprehensive LCICC also contributes to improving environmental quality. Integrating previous and current results, LCICC helps enhance regional ecological environment quality and high-quality development.

**Table 2:** Benchmark results

Independent variable	Dependent variable: carbon intensity		
LCICC	-0.501***		
	(-3.75)		
Innovation construction		-0.424***	
		(-3.37)	
Low-carbon construction			-0.404***
			(-3.56)
Foreign investment	-5.200*	-4.725**	-5.315**
	(-2.43)	(-2.19)	(-2.49)
Economy	-2.221***	-2.246***	-2.244***
	(-11.55)	(-11.47)	(-11.55)
Trading openness	-0.041	-0.060	-0.043
	(-0.53)	(-0.70)	(-0.54)
Population	-2.234***	-2.301***	-2.227***
	(-3.41)	(-3.52)	(-3.50)
Education	-0.120	-0.169	-0.079
	(-0.11)	(-0.15)	(-0.07)
Urbanization	0.203	0.147	0.094
	(0.27)	(0.19)	(0.13)
_cons	37.79***	38.44***	38.025***
	(9.26)	(9.23)	(9.47)
City fixed effects	Yes	Yes	Yes
Year fixed effects	Yes	Yes	Yes
N	4080	4080	4080
R^2^	0.510	0.511	0.512

T statistics in parentheses; * *p* < 0.05, ** *p* < 0.01, *** *p* < 0.001

### Robustness test

#### Parallel trend hypothesis test

We use the event study method to verify whether the research sample satisfies the parallel trend assumption, ensuring the validity of the DID model. The event study model is as follows:

3 $$\begin{aligned}& carbon\_intensity_{i,t} \\ &\quad = \beta_{0} + \mu_{i} + \lambda_{t} + \sum_{\tau=1}^{m} \beta_{-tau} did_{i,t-tau} + \beta did_{i,t} \\ & \qquad + \sum_{\sigma=1}^{n}\beta_{+\sigma} did_{i,t+\sigma} + \beta_{1} control_{i,t} + \varepsilon_{i,t} \end{aligned}$$

*t-τ* represents the impact *τ* periods before the experiment, and *t + σ* represents the impact *σ* periods after the experiment. When the time is the experimental period, *did* takes the value of 1; otherwise, it is 0.

**Fig. 2:**
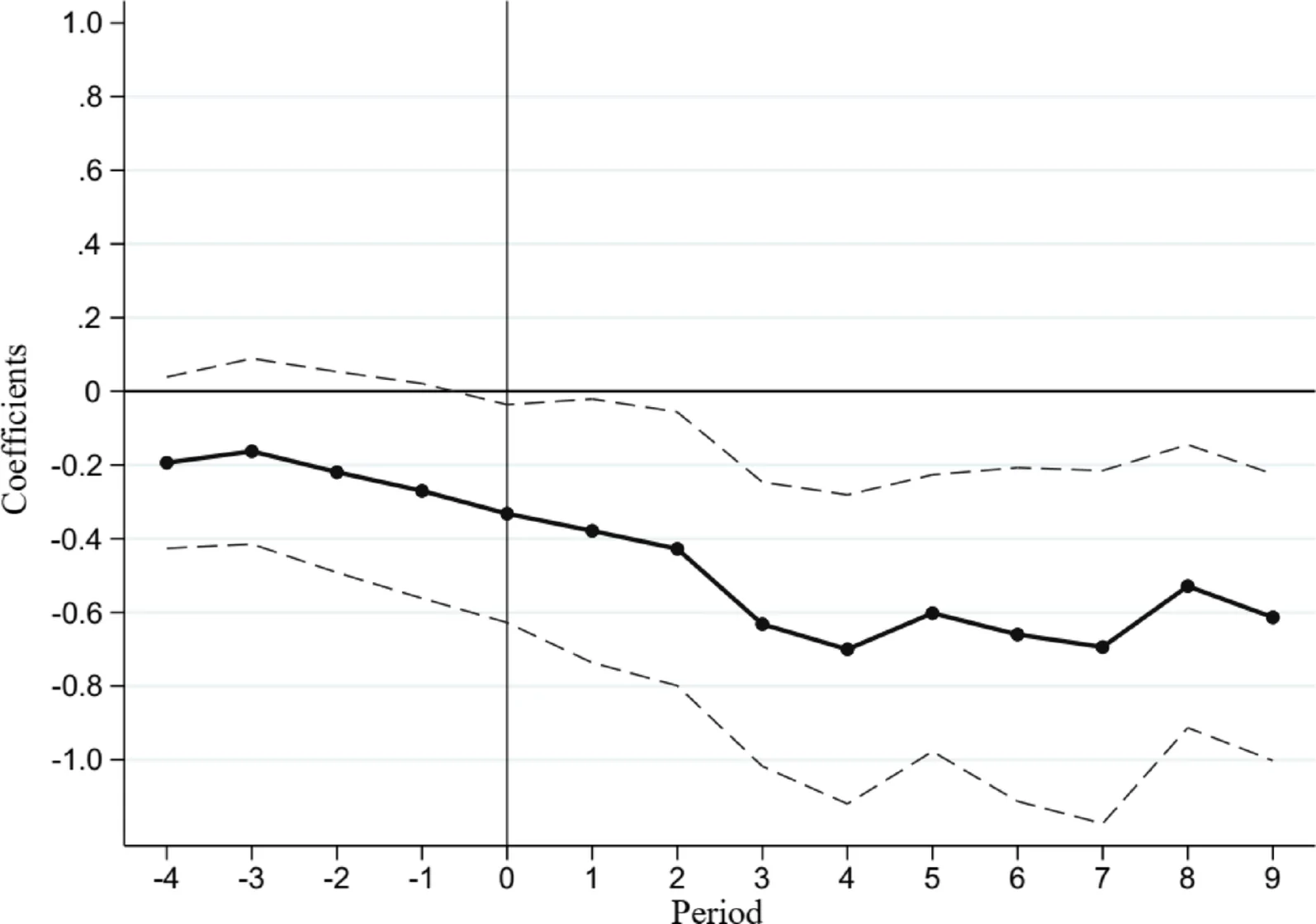
Common trend test

Figure 2 shows that before LCICC, there is no significant difference in carbon intensity between the treatment and control groups, and the results are statistically insignificant (confidence intervals include 0). After the transformation, the difference in carbon intensity between the treatment and control groups shows a fluctuating increasing trend, indicating that the carbon intensity reduction effect of LCICC pilot policies is continuously strengthening and statistically significant (confidence intervals do not include 0). Figure 2 demonstrates that the impact of LCICC on carbon intensity satisfies the parallel trend assumption.

#### PSM-DID estimation

Since the central government may prioritize cities with strong independent innovation capabilities, prominent technological leadership, high levels of sustainable socio-economic development, solid industrial foundations, and regional influence as pilot cities, these cities inherently have better foundations and trends for reducing carbon intensity, which may lead to selection bias and ultimately affect the benchmark regression results. To mitigate potential selection bias and enhance the validity of the control group, this study implements propensity score matching (PSM) methodology. Following established practices [63], the matching procedure utilizes the full set of control variables from Model (1) as matching covariates. A logit model is used for 1:1 nearest neighbor matching. The PSM balance test results (Table 3) show that before matching, all covariates exhibit significant differences. After matching, except for foreign investment, whose standardized bias absolute value reaches 23.3%, the standardized biases of all other matching variables are around 10% or smaller. Moreover, except for foreign investment, the mean differences of all covariates before and after matching change from significant to insignificant, indicating that the treatment group and the new control group are very close.

**Table 3:** Balance test

Variable	Unmatched	Mean		%bias	%reduct |bias|	t-test	
	Matched	Treated	Control			t	P>|t|
Foreign investment	U	0.026	0.018	45.5	48.9	4.81	0.000
	M	0.026	0.022	23.3		2.05	0.041
Economy	U	11.125	10.098	180.6	98.7	18.20	0.000
	M	11.078	11.064	2.3		0.26	0.794
Trading openness	U	0.538	0.178	79.6	89.2	11.51	0.000
	M	0.462	0.423	8.6		0.66	0.507
Population	U	6.719	5.667	117.4	96.8	13.34	0.000
	M	6.565	6.532	3.8		0.32	0.753
Education	U	0.164	0.183	-53.3	97.9	-5.25	0.000
	M	0.166	0.166	1.1		0.10	0.923
Urbanization	U	0.754	0.493	175.4	96.7	18.92	0.000
	M	0.735	0.743	-5.8		-0.50	0.620

Based on the matched sample, Model (1) is re-estimated. The empirical outcomes, presented in column 1 of Table 4, reveal that the PSM-enhanced difference-in-differences estimator maintains its statistically significant negative association, confirming that LCICC significantly reduces regional carbon intensity.

**Table 4:** Robustness test

Independent variable	PSM-DID estimation	Counterfactual test			Instrumental variable test^◊^	
	Carbon intensity				LCICC	Carbon intensity
LCICC	-0.362**					-7.649***
	(-2.72)					(-6.58)
LCICC _2		-0.427				
		(-1.19)				
Innovation_2			-0.324			
			(-1.56)			
Low-carbon_2				-0.356		
				(-0.81)		
Number of universities					0.050***	
					(9.56)	
Control variable	Yes	Yes	Yes	Yes	Yes	Yes
_cons	35.82***	37.58***	37.48***	37.87***	-0.298***	12.72***
	(7.87)	(9.12)	(9.03)	(9.38)	(-5.72)	(13.55)
City fixed effects	Yes	Yes	Yes	Yes	Yes	Yes
Year fixed effects	Yes	Yes	Yes	Yes	Yes	Yes
N	3125	4080	4080	4080	4080	4080
R^2^	0.515	0.509	0.509	0.511	0.117	0.470

T statistics in parentheses; * *p* < 0.05, ** *p* < 0.01, *** *p* < 0.001

“_2” indicates that the policy was implemented two years ahead of schedule

◊ Kleibergen-Paap rk LM = 90.64[0.000], Cragg-Donald Wald F = 176.62, Kleibergen-Paap Wald rk F = 91.36, Hansen J = 0.000

#### Counterfactual test

We construct the following counterfactual test to verify the robustness of the benchmark regression results. The implementation years of innovative or low-carbon pilot policies in each city are uniformly advanced by 2 years. If the *did* policy dummy variable remains significantly negative, it suggests that the reduction in carbon intensity may be due to other policies or random factors rather than LCICC. We consider three counterfactual scenarios: advancing both innovative and low-carbon pilot policies by 2 years, advancing only the low-carbon pilot policy by 2 years, and advancing only the innovative pilot policy by 2 years. The results are shown in Columns 2–4 of Table 4. Although the *did* coefficients are negative, they are statistically insignificant, indicating that the anticipated effects before LCICC do not significantly impact carbon intensity. Therefore, the reduction in carbon intensity is indeed caused by LCICC policies.

**Fig. 3:**
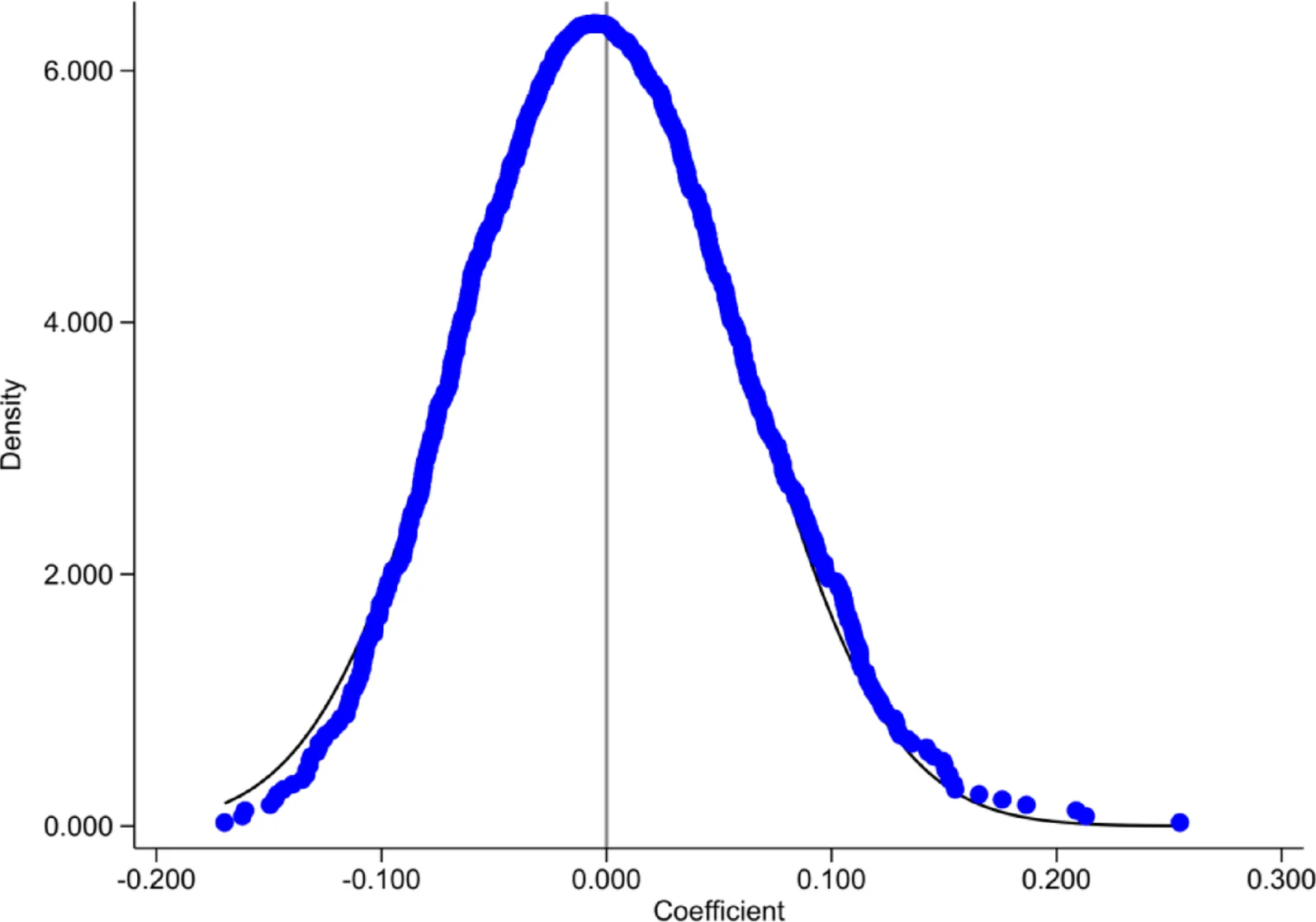
Placebo test

Another counterfactual design is the placebo test. Based on the innovative and low-carbon pilot status of 272 cities, we randomly select the same number of cities as the actual pilot cities in each year from 2010 onwards as the treatment group, with the remaining cities as the control group. We conduct 1,000 simulated estimations and obtain the estimated coefficients of the LCICC policy dummy variable in each regression. The kernel density plot of the estimated coefficients is shown in Fig. 3. The simulation results reveal that the coefficients from 1,000 iterations are normally distributed around zero. Notably, the distribution range of these coefficients significantly deviates from the benchmark estimate of -0.501 obtained from the actual regression analysis. This substantial discrepancy demonstrates that randomly assigned treatment groups fail to produce comparable carbon intensity reduction effects.

#### Endogeneity test

This paper further uses the instrumental variable method for robustness testing. The instrumental variable must satisfy two basic conditions: it must be correlated with the explanatory variable and uncorrelated with the residual term. This paper selects the number of universities as the instrumental variable for the policy dummy variable because it is related to both innovation and low-carbon development. A higher number of universities implies a greater advantage in human capital, indicating a higher level of innovation in the city. A higher number of universities also suggests better low-carbon technologies and a larger proportion of highly skilled talent, which facilitates the dissemination of low-carbon concepts. Therefore, cities with more universities are more likely to be included in the innovative and low-carbon city pilot lists. Additionally, the number of universities is often a historical legacy, related to the city’s location and historical status, but not directly related to carbon intensity, satisfying the exogeneity requirement. This paper uses the two-stage least squares (2SLS) method for instrumental variable testing, and the results are shown in Columns 2–6 of Table 4. The results show that the number of universities is significantly positively correlated with LCICC at the 1% level, consistent with expectations. Relevant statistical indicators show that the number of universities is not a weak instrumental variable. The second-stage estimation results show that LCICC significantly negatively affects carbon intensity at the 1% level. Therefore, after considering endogeneity, the benchmark findings in Table 2 remain robust.

### Influence mechanism

In the previous section, we confirmed that LCICC helps reduce regional carbon intensity. How does LCICC accelerate the reduction of regional carbon intensity? Are the channels hypothesized in the theoretical framework valid? This paper further verifies the potential mechanisms through which LCICC reduces carbon intensity by regressing each potential mechanism on LCICC. Table 5 shows that the positive effect of LCICC on technological innovation is significant at the 5% level, indicating a significant positive relationship between technological innovation, measured by patents, and LCICC. At the same time, technological innovation has been widely proven to help reduce carbon intensity [8, 37–38]. Therefore, LCICC significantly reduces carbon intensity through the technological innovation effect, supporting Hypothesis 1.

Column 2 demonstrates a statistically significant positive relationship between LCICC and industrial structure advancement. This structural shift toward service-oriented industries, as evidenced by previous studies [16, 55], contributes substantially to carbon intensity reduction. These results provide empirical validation for Hypothesis 3a. Column 3 reveals that LCICC significantly decreases the industrial structure rationalization index. Given the inverse relationship between the index value and actual rationalization level (as established in our methodology), this indicates a substantial improvement in industrial structure rationalization. Building upon existing literature that documents the carbon intensity reduction effects of industrial structure rationalization [46, 64], these findings support Hypothesis 3b.

In contrast, the expected effect of LCICC on environmental regulation is not significant (Column 4), indicating that local governments did not form stricter environmental constraints during innovative and low-carbon transformation, which is inconsistent with theoretical expectations. Hypothesis 4 is not supported. This null finding may stem from several factors. First, our measure, the frequency of environment-related terms in government work reports, captures policy rhetoric and agenda-setting, not actual enforcement intensity. Previous studies have documented a significant gap between stated environmental commitments and on-the-ground regulatory actions in Chinese cities [56]. Thus, the insignificant coefficient may reflect measurement error rather than the true absence of an effect. Second, LCICC involves multiple, sometimes conflicting, policy goals (economic growth, innovation, carbon reduction, social stability). Local governments may prioritize technologically feasible and economically beneficial measures (e.g. green finance, industrial upgrading) over stricter command-and-control regulations that could provoke business opposition [65]. Third, the multi-level governance structure of China’s environmental policy, where central government sets ambitious targets but local governments are responsible for implementation, can lead to strategic compliance or symbolic implementation [66]. Without credible enforcement threats, the actual intensity of environmental regulation may remain largely unchanged even as cities adopt low-carbon and innovative labels.

Finally, LCICC significantly enhances green finance. Furthermore, recent empirical study of Huang et al. [47] provided robust evidence supporting the significant role of green finance in facilitating carbon intensity mitigation. Therefore, Hypothesis 5 is supported.

**Table 5:** Influence mechanism test

Independent variable	Technological innovation	Industrial structure advancement	Industrial structure rationalization	Environmental regulation	Green finance
LCICC	0.048**	0.271***	-0.009**	-0.000	0.022***
	(2.64)	(4.61)	(-2.54)	(-0.97)	(4.31)
Control variable	Yes	Yes	Yes	Yes	Yes
_cons	-24.95***	-2.231*	0.474*	-0.007**	-0.815***
	(-18.17)	(-2.29)	(2.13)	(-3.07)	(-9.76)
City fixed effects	Yes	Yes	Yes	Yes	Yes
Year fixed effects	Yes	Yes	Yes	Yes	Yes
N	4080	4080	4080	4080	4080
R^2^	0.824	0.169	0.009	0.184	0.679

T statistics in parentheses; * *p* < 0.05, ** *p* < 0.01, *** *p* < 0.001

### Regional heterogeneity

Although the full-sample results show that LCICC helps reduce carbon intensity, can such a construction promote carbon intensity reduction in any region? Due to differences in historical conditions, resource endowments, and development stages, the paths and effects of economic and social development vary across regions [67]. Typically, the effectiveness of any policy is constrained by local socio-economic and environmental conditions [68]. Moreover, theoretically, it is difficult to design a universally applicable policy in a vast region like China, which has significant internal differences. Therefore, the study extends its analysis to investigate regional variations in the relationship between LCICC and carbon intensity. To capture potential geographical disparities, the analysis employs a regional disaggregation approach, stratifying the full sample into three distinct geographical clusters: eastern, central, and western regions. This classification aligns with China’s macro-level socio-economic development gradient, where the eastern region represents the most advanced economic zone, followed by the central region as an intermediate development area, and the western region as the emerging economic zone. The eastern region includes 82 cities; the central region includes 111 cities; and the western region includes 79 cities.

Besides, the study incorporates an administrative hierarchy perspective by classifying cities according to their political and economic significance. This classification scheme identifies provincial capitals and specially designated state-plan cities as high-tier administrative entities, while categorizing remaining municipalities as lower-tier administrative units. High-administrative-level cities are often regional socio-economic and cultural centers, with obvious advantages in transformation and development. In contrast, low-administrative-level cities have relatively scarce resources for transformation and development. The analysis concludes with an economic structure-based classification, distinguishing between resource-dependent and non-resource-dependent urban centers. Resource-dependent cities are dominated by natural resource extraction and processing, with urban production and development closely related to resource exploitation [49]. These cities often have higher levels of resource consumption and energy use and lower levels of innovation [9], rendering them particularly responsive to low-carbon transition policies and innovation-driven development strategies. Table 6 reports the heterogeneity test results for the above different types of cities.

**Table 6:** Regional heterogeneity test

Independent variable	High-tier	Lower-tier	Eastern	Central	Western	Resource	Non-resource
LCICC	-0.046	0.335**	-0.185	-0.752***	0.584	-0.376***	0.312**
	(0.108)	(0.149)	(0.113)	(0.232)	(0.369)	(0.116)	(0.126)
Control variable	Yes	Yes	Yes	Yes	Yes	Yes	Yes
_cons	25.322***	43.891***	21.866***	43.622***	44.981***	56.773***	32.947***
	(4.591)	(4.729)	(4.976)	(4.694)	(8.906)	(9.643)	(4.335)
City fixed effects	Yes	Yes	Yes	Yes	Yes	Yes	Yes
Year fixed effects	Yes	Yes	Yes	Yes	Yes	Yes	Yes
N	450	3630	1230	1665	1185	1350	2730
R^2^	0.701	0.515	0.566	0.632	0.457	0.624	0.462

Standard errors clustered to the city level in parentheses; * *p* < 0.1, ** *p* < 0.05, *** *p* < 0.01

The results show that although LCICC reduces carbon intensity in high-administrative-level cities, the effect is not statistically significant. In contrast, LCICC positively affects carbon intensity in low-administrative-level cities at the 5% significance level. Innovations (e.g. low-carbon technologies, green industries) typically diffuse from high-administrative-level cities (e.g. provincial capitals or first-tier cities) to low-administrative-level cities (ordinary cities) [31]. However, this process is characterized by time lags and unevenness. As regional political, economic, and cultural centers, high-administrative-level cities are often early adopters of innovative technologies. They have more resources, policy support, and market opportunities to promote low-carbon transformation. However, due to their larger economic scale and more complex industrial structures, the application of low-carbon technologies may focus more on incremental improvements (e.g. optimizing existing industries) rather than complete structural transformation [29]. Therefore, the effect of carbon intensity reduction may not be significant. Ordinary cities play a “follower” role in the innovation diffusion process. This dynamic is shaped by the “climate promotion tournament” mechanism, where local officials are incentivized to compete on carbon reduction performance under central mandates, but lower-tier cities often lack the administrative capacity and resources to respond effectively [69].They typically begin to introduce low-carbon technologies only after their successful application in high-administrative-level cities. However, ordinary cities have smaller economic scales and relatively simple industrial structures, and the introduction of low-carbon technologies may lead to a relative contraction of economic activity in the short term (e.g. the elimination of traditional high-carbon industries), resulting in an increase in carbon intensity. Furthermore, cities with elevated administrative status benefit from distinct advantages in fiscal capacity allocation, preferential policy access, and human capital accumulation, enabling them to more effectively promote low-carbon technological innovation and industrial upgrading. However, due to their large-scale economic activities, the effects of low-carbon transformation may be offset by economic growth, leading to insignificant reductions in carbon intensity. In contrast, ordinary cities have relatively insufficient resources and policy support, and their low-carbon transformation may rely more on external technology inputs and capital investments. This “passive transformation” may lead to a decline in economic efficiency in the short term (e.g. contraction of traditional industries and immaturity of emerging industries), thereby increasing carbon intensity.

The empirical findings reveal distinct regional patterns in the carbon intensity effects of LCICC. While central cities demonstrate statistically significant reductions in carbon intensity, the impacts in eastern and western regions present contrasting yet statistically insignificant trends. The eastern region represents China’s most advanced economic zone, with high urbanization levels and industrial structures dominated by services and high-tech industries. Since eastern cities are already at a high level of economic development, their innovative and low-carbon transformations are more about technological optimization and efficiency improvements rather than structural changes. Therefore, there is limited room for carbon intensity reduction. The central region is in the mid-stage of industrialization and urbanization, with industrial structures dominated by manufacturing and heavy industries. The low-carbon transformation of these cities is often accompanied by industrial structure adjustments and technological upgrades, which can significantly decrease carbon intensity. Additionally, industrial structures in eastern cities are dominated by services and high-tech industries, which inherently have lower carbon intensity. Therefore, the low-carbon transformation of eastern cities is more about marginal improvements (e.g. improving energy efficiency, promoting clean energy), with limited impact on carbon intensity. The industrial structures of central cities are dominated by manufacturing and heavy industries, which have higher carbon intensity. Through industrial structure adjustments (e.g. eliminating outdated capacity, developing emerging industries) and technological upgrades (e.g. promoting energy-saving technologies), central cities can significantly reduce carbon intensity.

Finally, LCICC significantly reduces carbon intensity in resource-dependent regions but significantly increases carbon intensity in non-resource-dependent regions. The resource curse theory suggests that resource-rich regions may inhibit the development of other industries due to over-reliance on resource-based industries, leading to a single economic structure and insufficient technological innovation. However, during urban innovation and low-carbon transformation, resource-based cities may significantly reduce carbon intensity through the optimization of resource-based industries. These cities are typically dominated by energy and mineral resource-based industries, which have higher carbon intensity. In the context of low-carbon transition, resource-dependent cities possess substantial potential for carbon intensity reduction through strategic optimization of resource utilization patterns. This can be achieved via dual pathways: (a) enhancing operational efficiency through clean energy technology adoption and energy efficiency improvements, and (b) systematically phasing out obsolete production capacities. Non-resource-based cities have economies dominated by manufacturing and services, which typically exhibit lower baseline carbon intensity. However, during transformation, non-resource-based cities may face the pains of industrial structure adjustment (e.g., contraction of traditional manufacturing, immaturity of emerging industries), leading to a decline in economic efficiency in the short term and thereby increasing carbon intensity.

## Conclusions and implications

The production and living agglomerations generated during urban expansion have made cities a major source of global carbon emissions. Promoting LCICC, industrial upgrading, energy structure optimization, and green technological innovation are key to sustainable development in cities worldwide [70]. This study makes a significant contribution by being the first to empirically isolate the synergistic carbon intensity reduction effect of jointly implementing innovation and low-carbon city pilots, an effect that exceeds the sum of individual policies. The practical significance include providing a cost-effective policy mix for resource-constrained cities, identifying market-based mechanisms (green finance) as more potent than command-and-control regulation, and offering actionable, region-specific strategies for China’s dual carbon goals and for other developing nations undergoing similar transitions. Using a sample of 272 Chinese prefecture-level cities from 2005 to 2019 and a time-varying difference-in-differences methodology, our findings show:

Both innovative and low-carbon transformations significantly reduce urban carbon intensity, but the impact is weaker than the cross-effect of innovative and low-carbon transformation. This means that comprehensive LCICC is more conducive to reducing carbon intensity than single-dimensional urban transformation, thereby more effectively promoting sustainable urban development. This is consistent with the synergy effect theory, which suggests that the synergistic effect of multiple transformation dimensions generates greater benefits. This synergy is not only reflected in the synergistic effect of technological innovation and low-carbon transformation but may also further reduce carbon intensity by optimizing resource allocation and enhancing policy coordination.

Our mechanism analysis shows that LCICC reduces carbon intensity primarily through three pathways: technological progress, industrial upgrading, and green finance development. However, the expected environmental regulation mechanism does not play a role. This indicates that for low-carbon development, transformation initiatives are effectively harnessing the innovation-driven technological effects, structural transformation effects, and financial resource allocation effects. The results indicate that market-oriented mechanisms, particularly green finance, coupled with technological progress, currently dominate urban low-carbon development, while the potential of administrative regulatory instruments remains underutilized at this developmental stage. This is consistent with the view of new institutional economy theory that market mechanisms and technological innovation can more effectively achieve low-carbon goals.

In terms of regional heterogeneity, LCICC significantly increases carbon intensity in low-administrative-level cities, while its inhibitory effect on carbon intensity in high-administrative-level cities is not significant. LCICC significantly reduces carbon intensity only in central cities and resource-dependent cities but increases carbon intensity in non-resource-dependent regions, meaning that the LCICC impact on carbon intensity is closely related to local industrial structure conditions and technological resource endowments. This is consistent with resource endowment theory and industrial structure theory. Low-administrative-level cities may experience increased carbon intensity during transformation due to limited resources and insufficient policy support, while central cities and resource-based cities have more significant transformation effects due to greater room for industrial structure adjustment.

In conclusion, this study establishes a novel conceptual framework and methodological approach for investigating the LCICC-carbon intensity nexus. The research findings highlight several promising directions for future inquiry, including: (a) in-depth examination of mechanism-specific pathways during LCICC processes, particularly the underlying causes of environmental regulation mechanism inefficacy and potential policy interventions for its revitalization; and (b) expansion of research scope to assess LCICC’s multidimensional impacts on broader sustainable development objectives, such as social equity enhancement and resource efficiency optimization. These research extensions would contribute to developing a more comprehensive theoretical foundation for sustainable urban development strategies, ultimately informing evidence-based policy formulation.

The main policy implications of this paper include three aspects. First, prioritize market-based instruments over command-and-control regulation. Our mechanism tests reveal that green finance significantly reduces carbon intensity, whereas environmental regulation (measured by policy rhetoric) does not. This contrast suggests that, in China’s current institutional context, financial incentives such as green credit, green bonds, and carbon pricing may be more effective than administrative mandates or symbolic regulatory commitments. While China has already established a comprehensive green finance policy framework, including the Green Finance Support Project Catalogue (2025 Edition) and national carbon emission trading, policymakers should further strengthen market-based tools by improving SMEs’ access to green finance and accelerating the standardization of transition finance. In contrast, relying solely on emission standards or inspection frequency without credible enforcement mechanisms is unlikely to yield additional carbon intensity reductions.

Second, leverage the synergy between innovation and low-carbon policies. Our benchmark results show that cities implementing both policies achieve greater carbon intensity reductions than those implementing only one. This synergistic effect operates through technology-push-pull complementarity and reinforced policy signaling. Rather than treating innovation and low-carbon goals separately, local governments should design integrated policy packages. For example, green patent subsidies could be linked to low-carbon performance metrics, and low-carbon pilot cities could be required to meet innovation benchmarks. Given that China has already launched multiple rounds of both pilot programs, the next step is to actively promote joint implementation and cross-policy coordination.

Third, differentiate strategies by city type based on heterogeneous effects. Our heterogeneity analysis finds that LCICC significantly reduces carbon intensity only in central and resource-based cities, while it unexpectedly increases carbon intensity in lower-tier and non-resource-based cities. Therefore, a one-size-fits-all approach is inappropriate. For central and resource-based cities, continuous industrial upgrading and technological retrofitting should be prioritized. For lower-tier and non-resource-based cities, policymakers should provide transitional support, such as skill retraining programs, green infrastructure investment, and fiscal compensation, to prevent short-term economic disruption before carbon intensity begins to decline. These differentiated recommendations go beyond generic regional strategies and directly address the heterogeneous outcomes we observed.

A notable limitation of this study concerns the environmental regulation mechanism. While we found no significant mediating effect, we cannot definitively conclude that LCICC fails to strengthen environmental regulation. Our proxy, text frequency in government work reports, captures policy rhetoric rather than actual enforcement. Future research should employ more direct measures of regulatory intensity, including the number of on-site environmental inspections, the total amount of pollution fines, the rate of environmental violation prosecutions, or the staffing and budget of local environmental protection agencies.

## Data Availability

The data supporting the findings of this study are available from the corresponding author, upon reasonable request. These data are not publicly available due to restrictions arising from ongoing collaborative research and the necessity for further analysis.
